# Dietary inclusion of *Asparagopsis taxiformis* significantly reduces methane emissions in dairy cows by mechanistically altering vitamin B12-dependent and other methanogenesis precursor pathways

**DOI:** 10.1186/s40168-026-02447-0

**Published:** 2026-06-12

**Authors:** Katie Lawther, Nicholas J. Dimonaco, Paul Donnelly, Abdulai Guinguina, Sophie J. Krizsan, Sharon A. Huws

**Affiliations:** 1 Institute for Global Food Security, School of Biological Sciences, Queen’s University Belfast, Belfast, UK; 2 Laboratory of Microbiology, Wageningen University, Wageningen, The Netherlands; 3 Production Systems, Natural Resources Institute Finland (LUKE), Jokioinen, Finland; 4 Department of Agricultural Sciences, Faculty of Applied Ecology, Agricultural Sciences and Biotechnology, Inland Norway University of Applied Sciences, Blæstad, Norway; 5 Department of Applied Animal Science and Welfare, Swedish University of Agricultural Sciences, Umeå, Sweden

**Keywords:** Asparagopsis, Methane, Ruminant, Rumen, Microbiome, Mode of action

## Abstract

**Background:**

Ruminant products are widely consumed due to their high protein and micronutrient content, but ruminant production contributes significantly to greenhouse gas emissions, with methane (CH₄) accounting for 33% of anthropogenic emissions. CH₄ is generated via fermentative processes by the rumen microbiome, primarily through hydrogen utilisation by methanogenic archaea. Feeding beef cattle the red seaweed *Asparagopsis taxiformis* (ASP) has been shown to reduce CH₄ emissions by up to 80%. However, the microbial mechanisms underlying this reduction remain poorly understood. In this study, Nordic Red dairy cows (122 ± 13.7 days in milk) were fed grass silage and concentrate (60:40 dry matter basis) either with or without 0.5% ASP (organic matter basis) in a Latin square design, and rumen fluid was collected 19 days into each of the 3 experimental periods.

**Results:**

ASP supplementation reduced CH₄ yield by 54% (g CH₄/kg DM). Metagenomic analysis revealed genes encoding pyruvate and propionate production pathways were more abundant in ASP treated animals, while those associated with acetate and CH₄ were reduced. Additionally, genes encoding vitamin B12 biosynthesis enzymes showed reduced abundances (e.g., adenosylcobinamide-GDP ribazoletransferase, EC 2.7.8.26, −29.92%). Vitamin B12 and its related cofactors are critical for methanogenic methyltransferases and C1 metabolism. Dominant taxa including *Prevotella* and *Methanobrevibacter* declined, while less abundant taxa increased their contribution to methane-related pathways, indicating niche displacement and community restructuring.

**Conclusion:**

ASP supplementation modulates the rumen microbiome through mechanisms extending beyond direct methanogen inhibition. The reduced abundance of genes involved in C1 metabolism and vitamin B12-dependent methanogenic processes suggest methane suppression is linked to broader restructuring of microbial metabolic networks. The redistribution of methane-related functions from dominant taxa to a wider taxonomic community indicates ecological reorganisation and functional resilience of the rumen microbiome. Collectively, these results reveal the multiple modes of action of ASP, establishing its promise as an effective methane mitigation strategy.

Video Abstract

**Supplementary Information:**

The online version contains supplementary material available at 10.1186/s40168-026-02447-0.

## Background

It is estimated that over 9.1% of humans do not have access to sufficient food and hence suffer from nutrient deficiencies and conditions such as anaemia and stunting [16]. This challenge is expected to intensify, as the world’s population is expected to reach 10.4 billion by the 2080 s [70], putting further pressure on agricultural, water, and land resources. Simultaneously, food production contributes up to 29.7% of global greenhouse gas (GHG) emissions [17], with agriculture playing a major role, mainly through livestock production and manure or slurry storage practices.

Ruminant products are widely consumed globally due to their high protein and micronutrient density. However, ruminant production is a major source of enteric methane (CH_4_), a potent GHG with 27–30 times the global warming potential (GWP) of carbon dioxide (CO_2_), albeit with a relatively short atmospheric lifespan [44]. Ruminants account for 33% of all anthropogenic CH_4_ emissions [11, 27]. CH_4_ is oxidised in the atmosphere, and has an approximately 12-year atmospheric lifetime, suggesting that reducing its emissions can have an immediate effect on global warming [65]. Consequently, a major humanitarian challenge is achieving food security, limiting malnutrition whilst also fulfilling global government climate legislation, including the Paris agreement to limit global warming to 1.5 °C above the pre-industrial baseline [72].

In ruminant animals, the rumen is the main fermentative compartment of the forestomach, hosting a complex and dynamic ecosystem including anaerobic bacteria, protozoa, fungi, archaea and viruses [46]. These microbes interact with each other and establish a symbiotic relationship with the host, providing it with energy in the form of volatile fatty acids arising from the breakdown of complex carbohydrates within dietary plant material [18, 60]. During this process, hydrogen is released and subsequently primarily utilised by methanogenic archaea to produce CH_4_, but studies have shown that this biochemical process can be disrupted without necessarily affecting the productivity of the animal [22, 34]. For example, efficient reductions in enteric CH_4_ emissions from ruminants have been achieved through innovative feeding strategies, such as the addition of the chemical 3-nitroxypropanol (3-NOP, Bovaer®) to the diet, resulting in approximately 25–30% reductions in CH_4,_ irrespective of the units used to express CH_4_ emissions [1, 22]. Additional works have shown that feeding macroalgae, particularly the tropical macroalga *Asparagopsis taxiformis* (*A. taxiformis,* ASP)*,* can reduce CH_4_ emissions from ruminants by between 55% and 65% (g/kg Dry matter intake (DMI)) in vivo when added to the diet of dairy cows [64], and at most reduce CH_4_ emissions by 98% (g/kg DMI) in vivo when added to the diet of steers [34], at an inclusion level of 0.2–0.5% of organic matter (OM).

To develop an effective anti-methanogenic dietary intervention, it is imperative that the mode(s) of action is known and that there are no detrimental trade-offs to the fermentative capacity of the rumen microbiome or overall animal health and productivity [3]. In terms of assessing the mode of action of *A. taxiformis* on the rumen microbiome (in vivo), few studies exist, and those do have mainly utilised amplicon-based metataxonomy to assess changes in bacterial and archaeal populations. For example, using the in vitro RUSITEC technology, which mimics the rumen environment, coupled with metataxonomy, Roque et al. [58] showed that *A. taxiformis* did not substantially alter the rumen microbiome. In contrast, O’Hara et al. [48] found a total reduction in all methanogenic archaea with increases in the bacterial genera *Prevotella*, *Bifidobacterium*, *Succinivibrio*, *Ruminobacter*, and an unclassified genus from the family *Lachnospiraceae*. Using in vivo studies, Krizsan et al. [35] noted that rumen *Prevotella* were reduced in abundance, and there was a switch from *Methanobrevibacter* as the dominant archaeal genus to *Methanomethylophilaceae*, following feeding lactating dairy cows 0.5% DMI of *A. taxiformis*. Using metataxonomic approaches, Indugu et al. [26] found that feeding *A. taxiformis* at 0.5% DMI to lactating dairy cows reduced the abundance of *Methanoshaeara*. Moreover, using shotgun metagenomics analysis, Indugu et al. [26] showed that feeding *A. taxiformis* reduced the copy number of the methyl-coenzyme M reductase (MCR) gene, which encodes the enzyme responsible for the final step in CH_4_ production by catalysing the incorporation of coenzyme M and coenzyme B, resulting in heterosulfide production and the release of CH_4_.

Although some data exists on the modes of action of *A. taxiformis,* findings are contradictory and remain inconclusive, highlighting the need for expanded exploration beyond taxonomy into functional changes. While taxonomical changes are important markers of microbiome health, understanding functional repertoires and their temporal dynamics may offer greater biological insight. Consequently, this study aimed to assess the effects of feeding *A. taxiformis* at 0.5% OM inclusion level on the rumen microbiome using shotgun metagenomics, thereby providing insights into the potential modes of action.

## Materials and methods

### Study design

This study complements the work undertaken by Krizsan et al. [35] and provides novel information on the mode of action of *A. taxiformis* in the rumen. As such, more in-depth information on the animal parameters can be found in Krizsan et al. [35]. In brief, the animal experiment was conducted at Röbäcksdalen experimental farm of the Swedish University of Agricultural Sciences in Umeå (63°45′N, 20°17′E), comprising three Nordic Red dairy cows weighing (mean ± SD) 611 kg ± 62.1 kg and were 122 ± 13.7 days in milk. The cows were kept in an insulated free-stall barn, offered a total mixed ration ad libitum, mixed with a feed mixer (Nolan A/S, Denmark). They had free access to drinking water and were milked twice per day, at 06:00 and 16:30, throughout the experiment, and fed in individual feed troughs three times per day at 07:00, 13:00, and 20:00. Cows were all multiparous and blocked according to pre-trial milk yield, calculated as the mean of the 10 days preceding the trial. Within blocks, cows had similar parity and days in milk (DIM), with average milk yields of approximately 35.3, 37.4, and 32.7 kg/day and DIM of 136, 114, and 126 days in blocks 1, 2, and 3, respectively.

The cows were randomly allocated to a dietary treatment within block, i.e., square, and assigned to an extra-period Latin square change-over design consisting of three equal squares, where treatments in the extra period are the same as in the last period of the Latin square [43]. The experimental periods lasted 21 days, with the last 7 days used for data recording and sampling. All cows were fed a control diet composed of 600 g/kg dry matter of grass silage, 390 g/kg DM of a commercial concentrate (Komplett Amin 180, Sweden), and 10 g/kg DM of mineral mix (Mixa Optimal, Sweden). Dietary treatments included either no supplementation (control, CNT) or supplementation with the macroalga *A. taxiformis* (ASP) at 0.5% of OM intake.

### Rumen sampling

Rumen fluid was collected and prepared following Chagas et al. [9]. Samples were consistently obtained after morning milking at 09.00 on day 19 of each experimental period (P1, P2, and P3) using a stomach tube (RUMINATOR, Germany), with the first ~ 500 mL discarded to minimise saliva contamination. Then, a sample of 500 mL was taken and filtered through a two-layer cheesecloth. Subsamples for microbial analysis were transferred to 2.0 mL Eppendorf tubes, immediately frozen using liquid nitrogen, and kept at − 80 °C until analysis.

### Rumen microbiome sequencing

DNA was extracted using a Qiagen DNeasy PowerSoil Pro Kit (Qiagen, Germany), following the manufacturer’s guidelines. Briefly, 500 µL of thawed rumen fluid was centrifuged at 15,000×*g* for 5 min, after which the supernatant was discarded. The pellet was then resuspended in 800 µL of CD1 solution (provided in the Qiagen DNeasy PowerSoil Pro Kit), vortexed briefly, and transferred to a PowerBead Pro tube (Qiagen, Germany). Mechanical cell disruption and homogenisation were performed through bead-beating at 5.5 m/s for 3 1-min cycles in a FastPrep instrument (MP Biomedicals, USA), with ice incubation between cycles. A 75-µL aliquot of ZymoBIOMICs Microbial Community Standard (D6300, Zymo Research, USA) was used as a positive extraction control, while a reagent-only control, ‘kitome’, was also included as a negative control; both controls were included in all downstream sequencing and analysis.

The concentration of DNA was quantified using both a NanoDrop One/OneC microvolume UV–Vis spectrophotometer (ThermoFisher Scientific, USA) and Qubit fluorometer (ThermoFisher Scientific, USA). The DNA underwent library preparation using the Illumina DNA Prep kit (Illumina, USA) with a DNA input of 250 ng. Thirty-eight samples underwent sequencing, with 3 animals at 3 time periods (technical replicates of 2) selected for each treatment and the positive and negative controls. Shotgun metagenomic sequencing was performed at the Queen’s University Belfast Genomic Core Technology Unit using an Illumina NovaSeq 6000 with an S4 300 flow cell (Illumina, San Diego, CA, USA). Paired-end sequencing was conducted with a read length of 150 base pairs.

### Rumen microbiome sequence analysis

Sequencing of the 36 rumen fluid samples produced a total of 2,084,236,576 raw reads, with an average of 57,895,460 reads per sample. FastQC (MultiQC v1.30 [15]) was used to generate an overall sequence quality and distribution report, which showed the reads had, on average, a PHRED score of ~Q36. Next, Trimmomatic (v0.39, [4] ) with the following parameters: ILLUMINACLIP: TruSeq3-PE-2:2:30:10 LEADING:3 TRAILING:3 SLIDINGWINDOW:4:20 MINLEN:50 was used to trim low-quality bases and adapters and identify reads with corresponding pairs (dropping reads if one pair was missing during sequencing). The reads were trimmed with a ‘SLIDINGWINDOW’ if at any site of 4 sequential bases the average quality dropped below Q20. Any reads reporting a quality score of < Q20 and short reads less than 50 bp (after trimming) were discarded.

The ‘cleaned’ reads from each of the samples were then mapped to the human (GCF_000001405.40_GRCh38.p14), cow (GCF_002263795.2_ARS-UCD1.3), and sheep (GCF_016772045.1_ARS-UI_Ramb_v2.0) reference genomes using Bowtie2 (v2.5.1, [37] with default parameters to identify the level of contamination and remove human and host-associated DNA. Reads that aligned with these reference genomes were considered contaminants and discarded. This resulted in a total of 1,956,850,528 clean reads, an average of 54,356,959 reads from each sample. These quality-checked and decontaminated reads were metagenomically assembled using metaSPAdes (spades v4.0.6 [47]). This resulted in 786,040 contigs with an average of ~ 21,834 per sample that were greater than or equal to 2500 bp in length. The contigs underwent taxonomic classification using Kraken2 (v2.1.3, [80]) using the ‘k2_pluspfp_20240904’ (Standard plus Refeq protozoa, fungi and plant) precomputed database with default parameters.

Additionally, Pyrodigal (v3.0.1, [25, 38] was used to predict protein-coding genes from the same contigs, which were subsequently annotated with eggNOG-mapper (v2.1.12, [8] using the eggNOG 5.0 database [23]. Bowtie2 was applied to map the cleaned reads back to the assembled contigs to count the number of reads assigned to each taxon that had been identified by Kraken2. Next, BEDtools (v2.31.1, [53]) intersect was used to identify reads that specifically mapped to protein-coding regions previously predicted by Pyrodigal on the assembled contigs. This step ensured that gene-level abundance estimates were captured regardless of differences in contig assembly. The MetaPont package (pypi.org/project/MetaPont/) was developed to process the outputs of this workflow and includes a collection of tools for merging and analysing results from the various steps, producing tab-separated value files for downstream interpretation. These files contained the full taxonomic and functional classifications alongside read mapping counts for each sample. A systematic extraction of the taxonomic contributors to functions of interest was then performed, providing insights into which taxa were driving key functional traits in each sample.

### Methanogen genome read mapping

To investigate the presence and species-level diversity of known rumen methanogens, organisms that are often low in abundance and challenging to reconstruct through metagenomic assembly, we performed read mapping against a curated set of 26 rumen-associated methanogen genomes, and 1 Gram-positive bacterium, *Syntrophomonas wolfei* subsp. *wolfei* strain Göttingen known to participate in syntrophic interactions with methanogens [39]. Reads were aligned using Bowtie2 with the very-sensitive-local parameter, allowing for greater sequence divergence between reads and the reference genomes. The number of reads mapping to each contig within each genome was recorded. Subsequently, BEDtools genomecov was used to calculate coverage statistics, generating a genome-wide coverage value that accounted for all contigs assigned to each methanogen genome. The ratio of the total number of mapped reads (to the genome) to the total number of reads per sample (MP:TR) was calculated for each genome. The complete metagenomic workflow is publicly available at github.com/TheHuwsLab/Metagenomic_Workflow*.*

### Statistical analysis and visualisation

The negative control was investigated for contamination, and the taxonomy assigned to the positive control was compared to the expected taxonomic distribution for the ZymoBIOMICs Microbial Community Standard. All analyses were performed in RStudio (v2024.9.1.394) (R version 4.4.2 (2024–10–31)) and visualised using ggplot2 (v3.5.1, [76] unless otherwise specified. To consider biases, including library size difference, samples (reads described above) underwent normalisation using the trimmed mean of M-values (TMM) ([52, 56, 67]) using the edgeR package (v4.4.1, [10] and the ‘calcNormFactors’ function. Following normalisation, bacterial and archaeal data were separated, and phyla and genus levels were selected for further analysis. Relative abundance was calculated for each domain at each level and visualised in stacked bar charts. All downstream statistical analyses were then conducted on five functional and taxonomic levels: archaeal genera, bacterial genera, KEGG pathways, enzyme commission (EC) number, and carbohydrate-active enzyme (CAZy) families.

Three alpha diversity indices were calculated using the vegan package (v2.6.8, [49]: Shannon diversity index, inverse Simpson index, and Chao1 richness estimator. Shannon and inverse Simpson indices were computed on normalised abundance data, while Chao1 was calculated on integer-rounded counts as required by the estimation algorithm. Effects of treatment (ASP vs. CNT) and experimental period on alpha diversity were assessed using linear mixed-effects models (LMMs) implemented with the lme4 (v1.1.37, [2] and lmerTest (v3.1.3, [36] packages. The model structure was: Diversity Metric ~ Treatment + Period + (1|Cow), where Treatment and Period were modelled as fixed effects and Cow was included as a random intercept to account for repeated measurements within the Latin square design. Models were fitted using restricted maximum likelihood (REML). Post-hoc pairwise comparisons were performed using estimated marginal means (emmeans, v2.0.1, [40] to evaluate (i) treatment differences within each period, and (ii) period differences within each treatment. *P*-values from pairwise tests were adjusted for multiple comparisons using the Benjamini–Hochberg (BH) method, with adjusted *P* < 0.05 considered statistically significant. Finally, alpha diversity indices, Chao1, inverse Simpson, and Shannon, were visualised in box plots.

Compositional beta-diversity was assessed using the Aitchison distance, calculated as the Euclidean distance between centred log-ratio (CLR)-transformed normalised read abundances. Features were retained for analysis if they occurred in ≥ 10% of samples and had ≥ 100 total reads across all samples. Differences in community composition among treatments and periods were tested using permutational multivariate analysis of variance (PERMANOVA; adonis2 function [45], vegan v2.6.8, [49] with 999 permutations. To account for the repeated-measures structure of the Latin square design, permutations were constrained within cow (permute::how, blocks = Cow). Marginal (Type III) tests were performed to assess the independent effect of each term. Pairwise post-hoc comparisons were conducted to identify specific treatment and period differences. Within each period, treatments were compared using PERMANOVA without blocking. Similarly, within each treatment, all pairwise period comparisons were tested without blocking. Again, *P*-values from pairwise tests were adjusted for multiple comparisons using the BH method, with adjusted *P* < 0.05 considered statistically significant. Ordination of compositional dissimilarity was visualised using principal coordinates analysis (PCoA) based on the Aitchison distance matrix.

To evaluate potential carryover effects inherent to the Latin square design, we tested whether microbiome composition in Periods 2 and 3 was associated with the treatment administered in the preceding period (PrevTrt). For each cow, PrevTrt was defined as the treatment received in the immediately preceding period (e.g., for Period 2, PrevTrt corresponds to the Period 1 treatment). Carryover effects were assessed using PERMANOVA on the Aitchison distance matrix, with the model: Distance ~ Treatment + PrevTrt + Period (PERMANOVA; adonis2 [45] function, vegan v2.6.8, [49]. Two PERMANOVA tests were performed: a standard test with 9,999 permutations and a high-precision test with 99,999 permutations. In both cases, permutations were constrained within cow to preserve the repeated-measures structure. Marginal tests were used to assess the independent contribution of each predictor. To distinguish between location and dispersion effects, we tested for homogeneity of multivariate dispersion (PERMDISP, betadisper and permutest functions, vegan v2.6.8, [49] separately within Period 2 and Period 3, examining dispersion differences among PrevTrt groups, current Treatment groups, and the four-level interaction (Treatment × PrevTrt cells) (vegan v2.6.8, [49].

Both linear discriminant analysis effect size (LEfSe) analysis was completed and LDA plots were visualised using the python scripts: lefse_format_input.py, lefse_run.py, lefse_plot_res.py, lefse_plot_features.py, using the lefse conda (v25.1.0) package ([v1.1.2], [63], SegataLab/lefse). Default parameters were utilised, and the option –o 1,000,000 was included as recommended in the authors’ manual. This scales the feature such that the sum (within the same taxonomic level) equals 1,000,000, which allows the LDA score to produce more meaningful values. This package includes an internal Wilcoxon test and outputs discriminative features with an LDA score > 2.0. Tests were performed using treatment as the class variable across the three periods (P1, P2, and P3), with read counts as subject vectors. All code used for statistical analysis and figure creation is publicly available at github.com/lawkj/Asparagopsis_Metagenomic_analysis.

## Results

A metagenomic approach was used here to investigate the effects of dietary supplementation with ASP on the dairy cattle rumen microbiome. The companion paper demonstrated that ASP supplementation reduced CH_4_ yield by 54% (g CH_4_/kg DM) and shifted volatile fatty acid molar proportions toward increased propionate (204 to 259 mmol/mol) and decreased acetate (629 to 552 mmol/mol), with corresponding changes in acetate-to-propionate ratios [35]. A total of 18 rumen samples were sequenced (in duplicate), representing two treatment groups (ASP-treated and control), with three animals per group sampled at three periods, alongside both a Zymo positive control and negative “kitome” control. The positive control showed the expected taxonomic profile consistent with the ZYMO mock community [Supplementary file 1], while the negative control yielded minimal reads numbering 428, 100% of which were UNCLASSIFIED [Supplementary file 1]. All taxonomic and functional results are based on the number of reads mapped to metagenomically assembled contigs, or their coding regions.

### Assessment of carryover effects revealed minimal influence on microbiome composition

No significant carryover effects were detected for archaeal, bacterial, CAZy, or KEGG functional profiles (*P* > 0.05 in all cases), with PrevTrt explaining approximately 4% of the total variance across data types. Enzyme Commission(EC) profiles showed a modest but statistically significant association with PrevTrt (PERMANOVA, *P* = 0.034), accounting for 3.9% of the variation. Tests of multivariate dispersion revealed no differences in within-group variability associated with PrevTrt in either Period 2 or Period 3 for any data type (PERMDISP, *P* > 0.10), indicating that the observed effects reflect differences in centroid location rather than heterogeneity of dispersion. Collectively, these results suggest that carryover effects were minimal in this study and unlikely to substantially influence treatment-related comparisons [Supplementary file 2].

### Shifts in bacterial genus-level associations and community complexity under *Asparagopsis* treatment

**Fig. 1:**
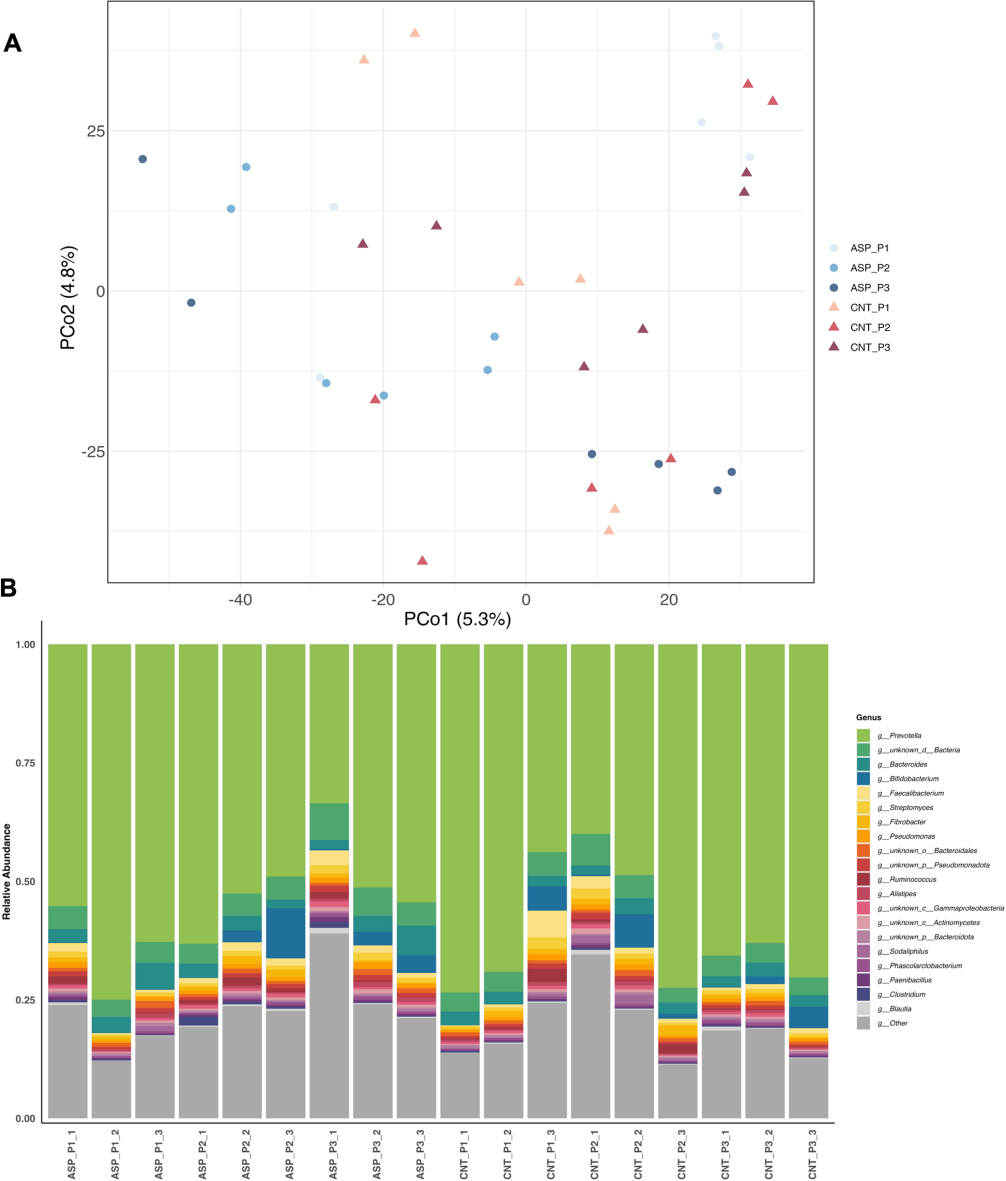
Bacterial community composition and diversity based on metagenomic reads mapped to contigs from rumen fluid DNA. **A** PCoA of bacterial genus-level community composition based on Aitchison distance matrices. Samples are labelled by treatment and period (e.g., ASP_P1, ASP_P2). **B** Relative abundance of the 20 most abundant bacterial genera across samples; remaining taxa are grouped as “Other”

On average, 82.93% of the contigs from each sample were annotated at the bacterial domain level, with a maximum of 88.11% and minimum 63.36% [Supplementary file 3]. To assess compositional differences, beta-diversity was evaluated using Aitchison distance, revealing that bacterial communities (at the genus level) were influenced by both treatment (adj. *P* = 0.001) and period (adj. *P* = 0.001) (Table 1). Pairwise comparisons showed that the bacterial microbiome under ASP differed significantly from CNT across all three periods (P1–P3: adj. *P* = 0.012, Table 1). Within-treatment comparisons across periods showed some temporal changes in ASP: P1 vs P2 (adj. *P* = 0.024) and P1 vs P3 (adj. *P* = 0.03), while no significant period-to-period differences were detected in CNT (Table 1). PCoA based on Aitchison distance revealed loose clustering of bacterial communities by treatment, with ASP samples separating from CNT samples primarily along PCo1 (5.3%) (Fig. 1A).

**Table 1:** PERMANOVA and pairwise PERMANOVA of bacterial and archaeal community composition based on metagenomic reads, at a genus level

		Adjusted *P*-values	
Comparison		Archaea	Bacteria
Treatment		0.003	0.001
Period		0.358	0.001
P1	ASP vs CNT	0.631	0.012
P2	ASP vs CNT	0.384	0.012
P3	ASP vs CNT	0.027	0.012
P1 vs P2	ASP	0.842	0.024
P1 vs P3	ASP	0.357	0.030
P2 vs P3	ASP	0.357	0.125
P1 vs P2	CNT	0.947	0.068
P1 vs P3	CNT	0.357	0.255
P2 vs P3	CNT	0.357	0.255

Pairwise tests compare treatments within each period and periods within each treatment. *P*-values were adjusted using the Benjamini–Hochberg method; significant values (adj. *P* < 0.05) are underlined

Alpha diversity was evaluated using Chao1 richness and the Shannon and inverse Simpson indices to capture complementary aspects of bacterial community diversity (Supplementary file 4). Neither treatment nor period significantly affected Shannon or inverse Simpson diversity (adj. *P* ≥ 0.05), indicating no detectable differences in community evenness. Across treatments and periods, average Shannon indices ranged from 2.25 to 3.28, and inverse Simpson indices ranged from 2.26 to 5.06, reflecting modest variation in evenness. In contrast, Chao1 richness differed significantly between treatments (adj. *P* = 0.011), with ASP samples consistently exhibiting higher richness (P1: 1011.83; P2: 1031.00; P3: 1049.83) compared to CNT samples (P1: 902.50; P2: 1015.00; P3: 918.67) across all periods (P1–P3: adj. *P* = 0.012; Supplementary file 4). No significant effects due to period were detected for Chao1 (adj. *P* = 0.252).

Considering the phylum level, the most dominant phyla across all treatments and time periods were Bacteroidota, Bacillota, and Pseudomonadota, with an average relative abundance of 66.74%, 10.65%, and 9.23%**,** respectively [Supplementary file 3]. The most abundant bacterial genus across all samples was *Prevotella*, irrespective of period and treatment, with an average relative abundance of 0.58 (58.0%). Followed by *Bacteroides* (average 0.03, 2.98%), *Bifidobacterium* (average 0.02, 2.26%) and *Faecalibacterium* (0.015, 1.50%) (Fig. 1B).

LEfSe analysis revealed two bacterial genera positively associated with control animals at P3: *Prevotella* and *Sphingobacterium* (adj. *P=* 0.004 and 0.010, respectively). In contrast, in ASP-treated animals, 59 genera were positively associated, including *Oscillibacter, Alistipes, Lachnoclostridium, Enterocloster*, and *Burkholderia* amongst others [Supplementary file 5].

### Rumen archaeal community composition and diversity responses to *Asparagopsis* supplementation

At the domain level, there was an average of 137,517 reads assigned to contigs that were reported as archaea, with an average relative abundance of 1.35% of the data. The maximum reads per sample assigned to archaea was 753,711 (relative abundance of 4.84%), while the minimum per sample was only 6401 (relative abundance of 0.06%) [Supplementary file 3].

**Fig. 2:**
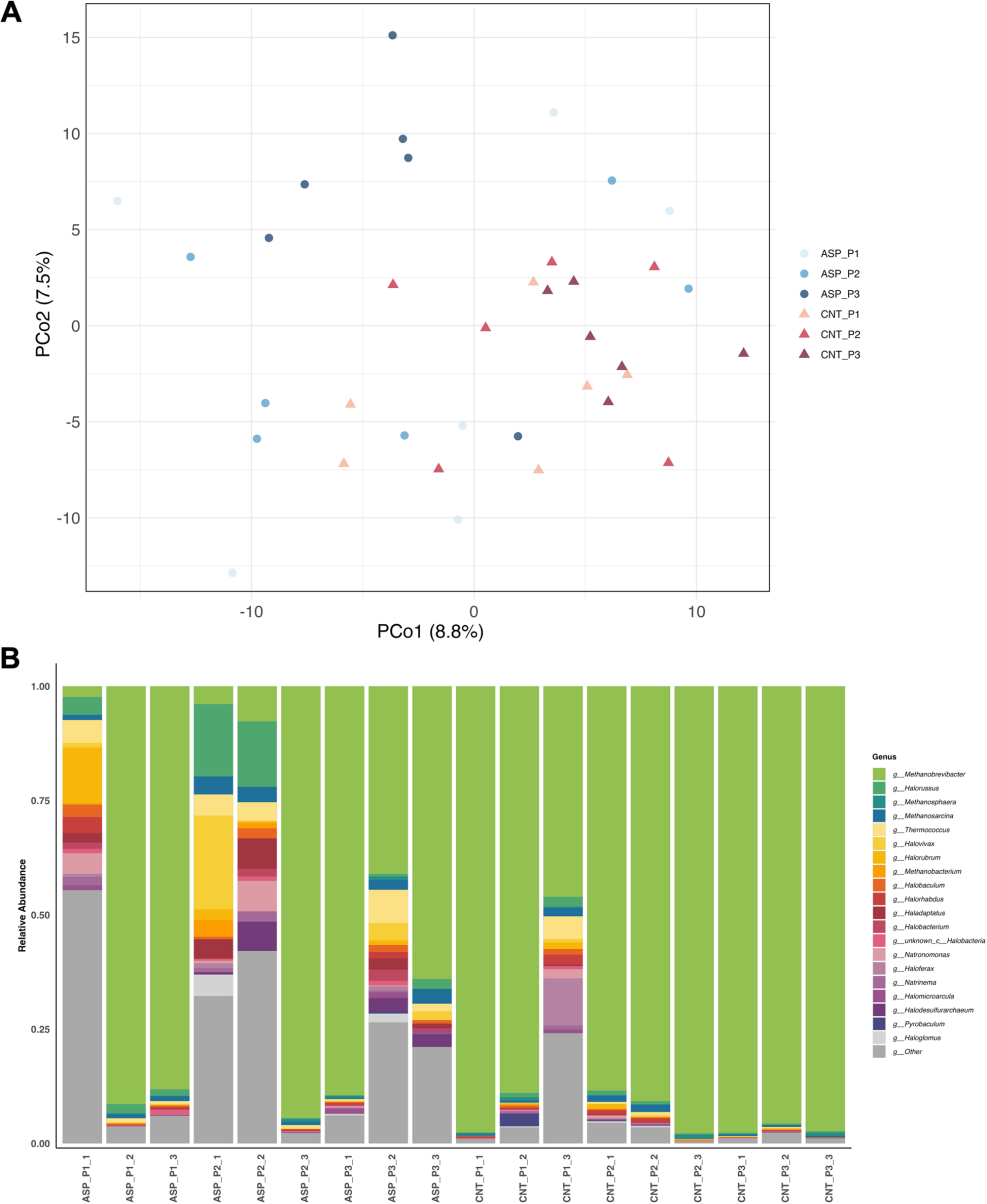
**A**, **B** Archaeal community composition and diversity based on metagenomic reads mapped to contigs from rumen fluid DNA.** A** PCoA of archaeal genus-level community composition based on Aitchison distance matrices. Samples are labelled by treatment and period (e.g., ASP_P1, ASP_P2) **B** Relative abundance of the 20 most abundant archaeal genera across samples; remaining taxa are grouped as "Other"

Compositional variation within archaeal communities was assessed at the genus level using Aitchison distance. Overall, treatment significantly influenced archaeal community composition (PERMANOVA, adj. *P* = 0.003), whereas period had no significant effect (adj. *P* = 0.358) (Table 1). Pairwise comparisons between ASP and CNT revealed a significant difference only in period 3 (P3; adj. *P* = 0.027). Within-treatment comparisons across periods revealed no significant temporal changes in archaea for either ASP or CNT adj. *P* ≥ 0.05 (Table 1). PCoA based on Aitchison distance indicated partial separation of archaeal communities by treatment along PCo1 (8.8%), although substantial overlap was observed across periods (Fig. 2A). Consistent with the previously reported pairwise differences, the ordination pattern showed separation between ASP and CNT only in period 3, with no significant temporal structuring observed within treatments (Fig. 2A).

Alpha diversity of archaeal communities was significantly influenced by treatment, with Shannon, inverse Simpson, and Chao1 indices showing treatment effects (adj. *P* = 0.003, 0.003, 0.025, respectively), whereas no significant effects of period were detected (adj. *P* ≥ 0.05) [Supplementary file 4]. Pairwise comparisons between ASP and CNT were significant across all periods for all three indices (Shannon: P1–P3 adj. *P* = 0.004; inverse Simpson: P1–P3 adj. *P* = 0.0035; Chao1: P1–P3 adj. *P* = 0.031), indicating consistently higher alpha diversity under ASP. Across treatments, average Shannon indices ranged from 0.25 to 1.96, and inverse Simpson indices ranged from 1.08 to 7.71, reflecting modest evenness overall. Chao1 richness was also consistently higher in ASP (P1: 29.33; P2: 27.67; P3: 30.67) compared to CNT (P1: 24.00; P2: 26.33; P3: 24.33), highlighting greater archaeal richness under ASP treatment [Supplementary file 4].

At the phylum level, taxonomic classification of archaea was high, with an average of only 0.003% relative abundance assigned as ‘unknown phylum’ [Supplementary file 3]. The majority of archaeal sequences belonged to Euryarchaeota, with an average relative abundance of 95.75%. The next most abundant phylum was Thermoproteota, although its average relative abundance across samples was low (2.27%) [Supplementary file 3]. At the genus level*, Methanobrevibacter* was the dominant genus in most samples. For example, over 95% of the microbiome in control treatment samples was composed of *Methanobrevibacter* at P3 (Fig. 2B).

**Fig. 3:**
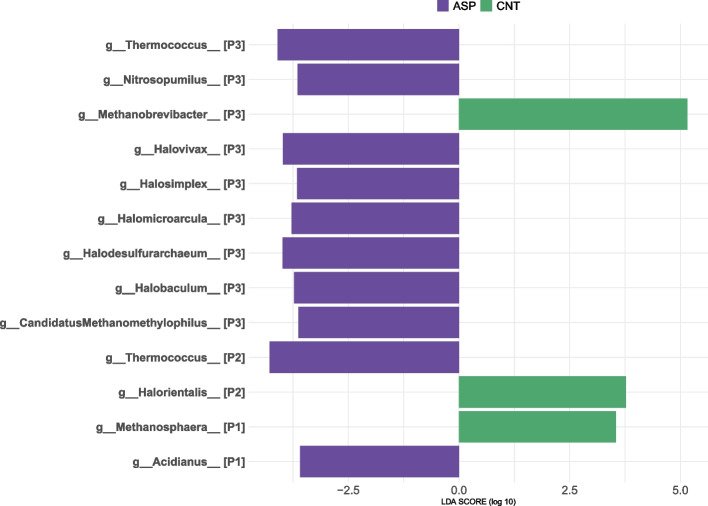
Differentially abundant archaeal genera identified using LEfSe (linear discriminant analysis effect size), associated with ASP and CNT treatments across three periods. Only archaea with significant association with a period are shown on the logarithmic LDA scores plot (Wilcoxon test and outputs discriminative features with abs LDA score > 2.0). Colours indicate the treatment group each archaeal genus is associated with based on LDA scores. Purple represents association with CNT animals, and green represents association with ASP-treated animals

Exploring the archaeal community further, linear discriminant analysis (LDA) revealed significant differences (Wilcoxon test and outputs discriminative features with abs LDA score > 2.0) between ASP and CNT treatments within each period (Fig. 3). At period 1 (P1), one archaeal genus was associated with each treatment (adj. *P* 0.042 and 0.022, respectively): *Methanosphaera* was significantly associated with CNT, and *Acidianus* was significantly associated with ASP, but neither was significantly linked to a treatment at either of the later periods (P2, P3). Focusing on P3, eight archaeal genera were significantly associated with the ASP treatment (adj. *P* < 0.05, Supplementary file 5), including halophiles, *Thermococcus* (which was also significant at P2) and the methanogen *Candidatus Methanomethylophilus*. In contrast, only one archaeal genus, *Methanobrevibacter*, was significantly associated with the CNT treatment at P3 (adj. *P* 0.022).

### Assembly-independent read mapping reveals species level archaeal diversity in the rumen microbiome

**Fig. 4:**
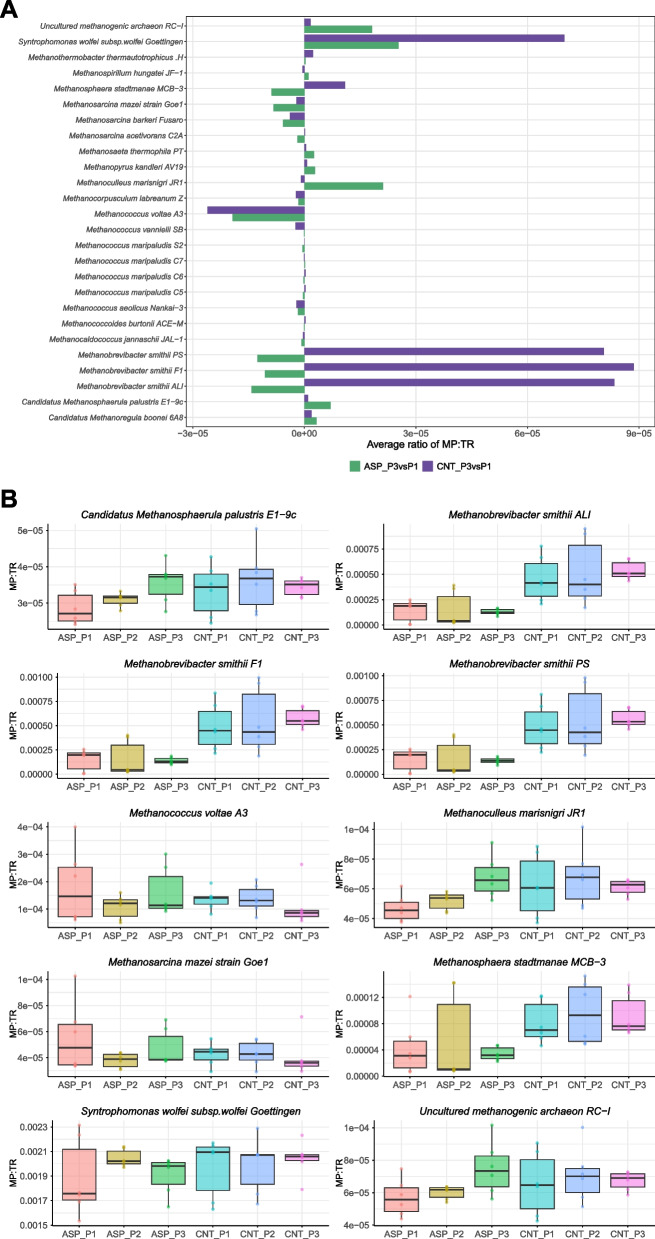
**A** Changes in relative abundance of 27 reference genomes across treatments and periods, measured as the ratio of mapped reads to total reads (MP:TR). Bars show the change in average MP:TR from P1 to P3, averaged across animals; purple indicates CNT and green indicates ASP. **B** Temporal dynamics of selected genomes with the largest changes in abundance. MP:TR ratios are shown across all periods using box-and-whisker plots. Points represent individual samples, boxes the interquartile range, and violins the distribution across replicates

To further explore the methanogen population diversity at a species-level, we employed a read-mapping approach. Using a collection of 26 methanogen genomes, we calculated the total mapped reads (to the methanogen genome) compared to the total number of reads per sample (MP:TR). We then investigated how the abundance of the methanogens changed throughout the study. This included 26 archaea species and 1 methanogen associated bacterial species *(Syntrophomonas wolfei* subsp. *wolfei* Goettingen) (Fig. 4A). When comparing P3 vs. P1, the genomes with the largest differences over time were then explored further (Fig. 4B).

In ASP-treated animals, *Methanoculleus marisnigri* JR1 increased in relative abundance over time. Additionally, an 'uncultured methanogenic archaeon (RC-1)' increased more substantially in ASP animals compared to CNT. Whereas *Methanobrevibacter smithii* (3 genomes) showed a modest decrease in ASP, as did *Methanosphaera stadtmanae* MCB-3, though this trend was variable, particularly in ASP_P2 (Fig. 4A, B). In CNT-treated animals, *Methanobrevibacter smithii* showed an overall increase in relative abundance over time. In contrast to ASP, *Methanosphaera stadtmanae* MCB-3 increased in CNT samples. The only bacterial genome included in the analysis, *Syntrophomonas wolfei* subsp. *wolfei* Goettingen, increased more markedly in CNT animals than in ASP, although its abundance varied between individual animals (Fig. 4A, B). Finally, *Methanococcus voltae* A3 decreased over time in both treatments at a similar rate.

### *Asparagopsis* treatment alters the abundance of genes encoding enzymes linked to methanogenesis and its precursor pathways

Three functional categories were examined: KEGG Pathways, EC numbers, and CAZy enzyme families [Supplementary file 3]. Treatment significantly influenced all functional categories, including CAZy (*P* = 0.002), EC (*P* = 0.002), and KEGG (*P* = 0.006), while period also had a significant effect across all categories (CAZy *P* = 0.009, EC *P* = 0.002, KEGG *P* = 0.013) (Table 2). Pairwise comparisons between ASP and CNT showed significant differences in CAZy across all periods (adj. *P* P1: 0.021, P2: 0.015, P3: 0.017) and in EC in P2 (adj. *P* = 0.045) and P3 (adj. *P* = 0.009), whereas KEGG pathway profiles did not differ significantly between treatments in any period (Table 2). Within-treatment comparisons across periods indicated some temporal changes: in ASP, CAZy differed between P2 and P3 (adj. *P* = 0.039), and in CNT, CAZy differed between P1 and P2 (adj. *P* = 0.039). No other significant temporal/period changes were detected (Table 2), with only loose clustering observed due to treatment in PCoA [Supplementary file 6].

**Table 2:** PERMANOVA and pairwise PERMANOVA of microbial functional profiles (CAZy, EC, KEGG Pathways)

		Adjusted *P*-values		
Comparison		CAZy	EC	KEGG pathway
Treatment		0.002	0.002	0.006
Period		0.009	0.002	0.013
P1	ASP vs CNT	0.021	0.103	0.408
P2	ASP vs CNT	0.015	0.045	0.132
P3	ASP vs CNT	0.017	0.009	0.408
P1 vs P2	ASP	0.362	0.072	0.084
P1 vs P3	ASP	0.125	0.072	0.374
P2 vs P3	ASP	0.039	0.072	0.374
P1 vs P2	CNT	0.039	0.121	0.374
P1 vs P3	CNT	0.204	0.121	0.374
P2 vs P3	CNT	0.068	0.121	0.374

Pairwise tests compare treatments within each period and periods within each treatment. *P*-values were adjusted using the Benjamini–Hochberg method; significant values (adj. *P* < 0.05) are underlined

Alpha diversity of the functional microbiome showed both treatment- and period-dependent patterns across Shannon, inverse Simpson, and Chao1 indices [Supplementary file 4]. Diversity of CAZy enzymes was influenced by treatment, with ASP consistently showing slightly higher richness and evenness than CNT across periods (Chao1 57.0–60.8 vs. 55.2–59.3; Shannon 3.28–3.30 vs. 3.23–3.29; inverse Simpson 18.69–18.88 vs. 17.48–18.68; pairwise P1–P3 adj. *P* ≤ 0.012). In contrast, EC functions varied primarily across periods rather than treatment, with richness and evenness slightly increasing over time (Chao1 1269–1294; Shannon 6.28–6.36; inverse Simpson 287–308), consistent with significant period effects (Shannon adj. *P* = 0.001; inverse Simpson adj. *P* = 0.002; Chao1 adj. *P* = 0.026) [Supplementary file 4].

**Fig. 5:**
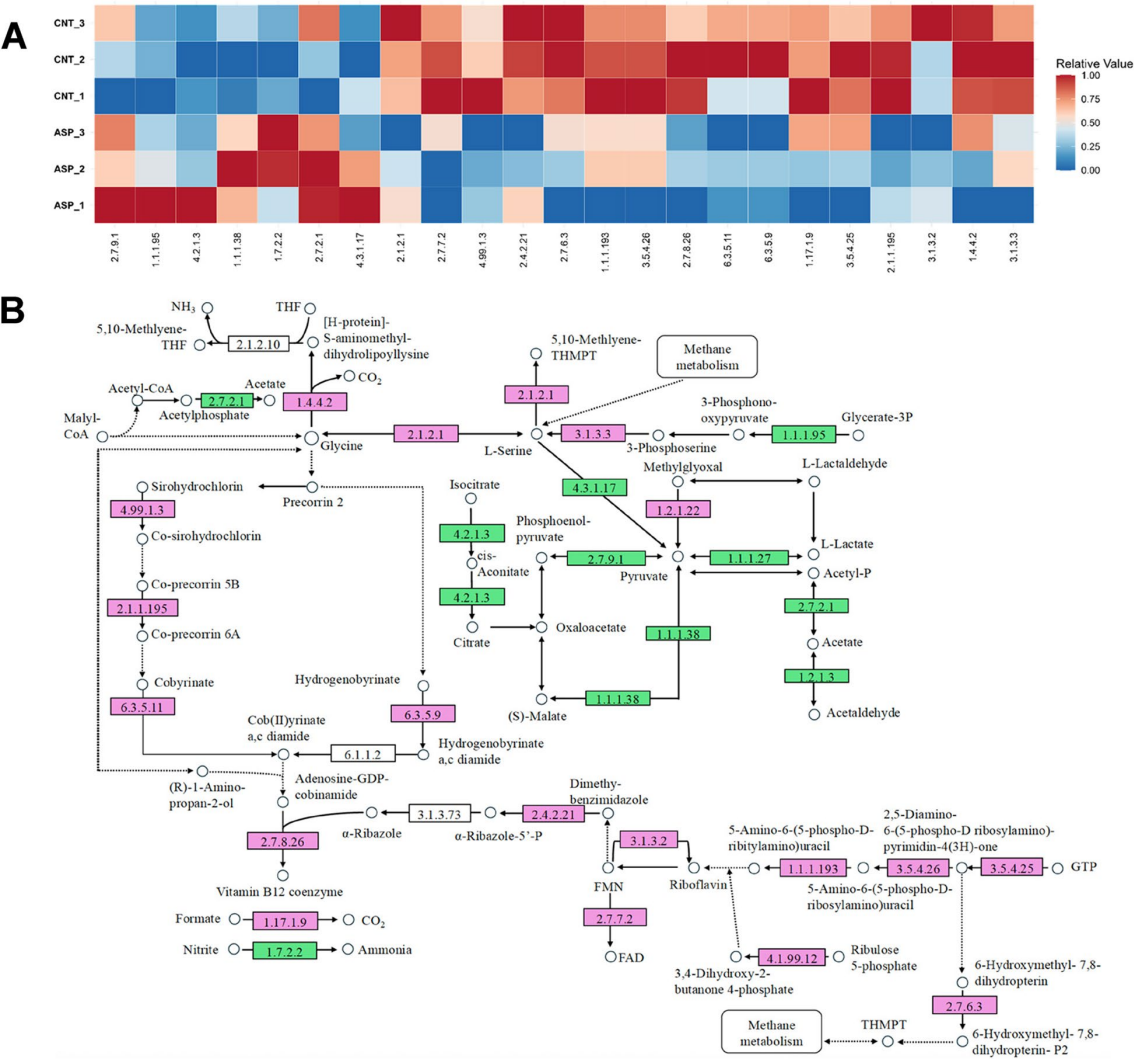
**A** Min–max scaled (0–1) normalised read counts for genes annotated as ECs involved in methanogenesis and precursor pathways. Only ECs significantly different as determined by LEfSe are shown. Full LDA scores for KEGG Pathway, EC, and CAZy are provided in Supplementary file 5. **B**: Methane-related pathways with ECs significantly associated at P2 and/or P3 (LEfSe). Pink indicates association with CNT and green indicates association with ASP

Twenty-seven enzymes that were significantly different at P2 and P3, as identified by LEfSe analysis, were further examined for their association with treatment and their potential interaction with CH_4_ metabolism [30]. Several genes encoding enzymes related to vitamin B12-dependent pathways were significantly more abundant in CNT diets than ASP diets (Fig. 5A and B, adj. *P* ≤ 0.05, Supplementary file 5). These included enzymes involved in glycine and L-serine metabolism, as well as GTP conversion, which is linked to tetrahydromethanopterin (THMPT) and vitamin B12 coenzyme formation, both with links to methanogenesis [30] (Fig. 5A, map00260, map00670, map00860, map00680). Vitamin B12 is essential for enzymes, such as methylmalonyl-CoA mutase and methionine synthase, that are often linked to one-carbon (C1) metabolism and methyl group transfers. Enrichment of genes encoding enzymes involved in glycine and L-serine metabolism suggests increased C1-unit generation, which feeds into folate and tetrahydromethanopterin (THMPT)-dependent pathways, key intermediates in methanogenic archaea.

The enzyme glycine dehydrogenase (EC 1.4.4.2), which mediates glycine cleavage to release CO₂, was notably elevated in control animals (Fig. 5A, B). CO₂ acts as the terminal electron acceptor in hydrogenotrophic methanogenesis, highlighting the enzyme’s potential role in supporting methane production. Additionally, the genes encoding the enzyme mediating formate conversion to CO₂ were also elevated (1.17.1.9), supporting increased C1 metabolism and methane-related pathways in the CNT group. This enzyme is crucial in hydrogenotrophic methanogenesis, especially in anaerobic archaea and some bacteria, where formate serves as an electron donor for methane production. Formate dehydrogenases are also important in syntrophic bacteria that provide substrates (H₂, CO₂) for methanogens.

In contrast, ASP-treated animals showed increased abundance of genes that encoded the enzyme involved in acetyl-CoA conversion to acetate (2.7.2.1), suggesting a shift in carbon flow away from methane-linked processes (Fig. 5A, B). Multiple genes encoding enzymes involved in a number of pathways leading to pyruvate production were also more abundant in ASP-treated animals, including those converting L-lactate and isocitrate to pyruvate. Furthermore, elevated abundances of enzyme-encoding genes able to produce pyruvate from acetate were observed, indicating a metabolic reprogramming toward energy production pathways less associated with methane synthesis (Fig. 5A, B). There was no significant difference observed for any of the comparisons for MCR (EC 2.8.4.1), the enzyme that catalyses the final stage of methanogenesis.

### *Asparagopsis* treatment drives diversification of taxa contributing to methane associated pathways

**Fig. 6:**
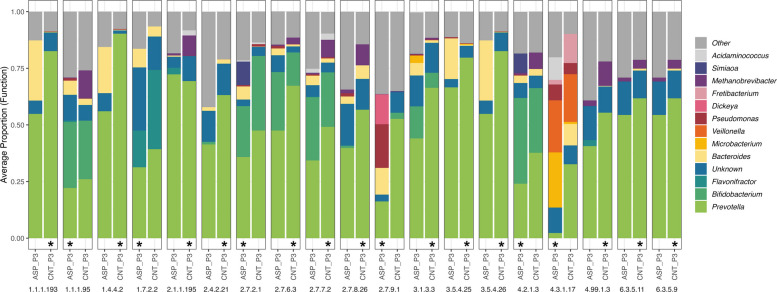
Stacked bar chart showing the average proportion of functions performed by taxa at P3. Bars represent the proportion of reads mapped to contigs from each taxon carrying the indicated functional (EC) ID. Only ECs significantly associated with treatment or control groups and linked to methane metabolism are shown. Taxa contributing < 10% of the total are grouped as “Other.” Asterisks denote significant associations with ASP or CNT at P3 (LEfSe)

MetaPont was used to extract genus-level taxonomic functional information, quantified as the proportion of reads assigned to contigs from a given taxon carrying a specific functional ID within each sample, with values expressed as averages across animals and treatments (Fig. 6, Supplementary file 7). Analysis of functional assignments revealed clear differences in taxa harbouring genes encoding enzymes involved in CH₄ production and feeder pathways between CNT and ASP animals by the end of the trial (P3) (Fig. 6). In the porphyrin and vitamin B12 biosynthesis pathway, genes encoding multiple enzymes were enriched in CNT animals, including cobalt-precorrin-5B (C1)-methyltransferase (EC 2.1.1.195), phosphoribosyltransferase (EC 2.4.2.21), adenosylcobinamide-GDP ribazoletransferase (EC 2.7.8.26), sirohydrochlorin cobaltochelatase (EC 4.99.1.3), and cobyrinate a,c-diamide synthase (EC 6.3.5.11). Across these functions, ASP animals consistently showed reduced contributions from *Prevotella* and *Methanobrevibacter*. For example, in EC 2.1.1.195, *Methanobrevibacter* proportionally decreased from 9.0% in controls to 0.9% in ASP, while other taxa such as *Starkeya* (1.5% CNT vs. 7.1% ASP), *Paracoccus* (0.5% vs 3.8%), and *Enterocloster* (undetected vs 3.5%) became more prominent in ASP animals. Similarly, for EC 2.4.2.21, *Prevotella* proportionally declined from 63.2% in controls to 41.4% in ASP, while *Bacillus* (0.3% CNT vs 5.9% ASP) and *Candidatus* Cloacimonas (0.1% vs 4.1%) were increased in ASP animals. For EC 4.99.1.3, reductions in *Prevotella* (55.4% CNT vs. 40.6% ASP) and *Methanobrevibacter* (2.5% CNT vs 0.8% ASP) in ASP animals were coupled with increases in *Dialister* (4.0% vs 7.9%) and *Chordicoccus* (0.1% vs 6.4%) (Fig. 6).

Within the riboflavin pathway, both 5-amino-6-(5-phosphoribosylamino)uracil reductase (EC 1.1.1.193) and diaminohydroxyphosphoribosylaminopyrimidine deaminase (EC 3.5.4.26), in CNT animals, were almost entirely attributed to *Prevotella* (> 80%). In ASP animals, however, *Prevotella* dropped to 55% while *Bacteroides* proportion was 27% (0.1% in CNT) (Fig. 6).

In the CH₄ pathway (map00680), phosphoglycerate dehydrogenase (EC 1.1.1.95) was carried primarily by *Prevotella* (26.0% CNT vs 22.1% ASP), *Bifidobacterium* (25.8% CNT vs 29.2% ASP), and *Methanobrevibacter* (12.5% CNT vs 0.4% in ASP). While, acetate kinase (EC 2.7.2.1) was associated with ASP animals and phosphoserine phosphatase (EC 3.1.3.3) associated with CNT animals. For acetate kinase, *Prevotella* and *Bifidobacterium* remained prevalent but decreased relative to control animals, while the candidate genus *Simiaoa* increased (10.4% in ASP, undetected in controls); this genus was first proposed by Liu et al. [42] but is not yet validly published under the International Code of Nomenclature of Prokaryotes. For phosphoserine phosphatase, ASP animals again showed reduced *Prevotella* (66.4% in CNT vs 44.0% ASP) with small but notable increases in *Bacteroides* (1.1% CNT vs 5.5% ASP) and *Microbacterium* (absent in controls vs 3.3% ASP) (Fig. 6).

As previously mentioned, *Prevotella* was significantly associated with CNT animals (adj. *P* = 0.004); overall, *Prevotella’s* contribution to the functions listed here decreased under ASP treatment. However, this decrease was not observed for all enzymes, for example, in the case of EC 2.1.1.195, there was no substantial difference between the *Prevotella* contributions in ASP animals (72.3%) and in CNT animals (69.3%). Overall, reductions in *Prevotella* and other dominant taxa such as *Methanobrevibacter*, due to ASP treatment, were accompanied by increases in *Bacteroides* and the emergence of additional taxa, including *Simiaoa, Starkeya, Paracoccus, Enterocloster, Dialister, Chordicoccus, Faecalibacterium*, and *Candidatus Cloacimonas* (Fig. 6). Functional contributions under ASP were distributed across a broader range of taxa compared to CNT, where functions were concentrated in a few dominant taxa.

## Discussion

This study examined the impact of ASP supplementation at 0.5% OM on the rumen microbiome of dairy cows in vivo, comparing ASP-supplemented animals to unsupplemented controls using metagenomic analysis. Feeding ruminants ASP at this level has been shown to reduce CH₄ emissions by up to 80% [32, 59], yet the mode of action on the rumen microbiome remains incompletely understood. Here, we provide evidence of ASP-associated differences in microbial gene abundances related to vitamin B12 biosynthesis, C1 and nitrogen metabolism, and methanogenesis precursor pathways, but not in the core methanogenesis pathway itself.

Other studies have suggested ASP achieves CH₄ reduction through inhibition of the MCR enzyme, similar to the mode of action of 3-NOP, a widely used methane inhibitor [26, 41]. However, our findings indicate that the reduction in CH₄ emissions following ASP supplementation is multifactorial and cannot be attributed to changes in genes associated with inhibition of a single enzymatic step in methanogenesis. Here, we observed a substantial reduction in CH₄ intensity [35] despite no significant change in the abundance of MCR, alongside changes in the abundance of genes encoding enzymes that are involved in the pathways of vitamin B12 and C1 metabolism. Vitamin B12 is an essential cofactor for several methanogenesis-related enzymes, including methyltransferases and methylmalonyl-CoA mutase, as well as enzymes involved in methionine remethylation within one-carbon metabolism and glycine cleavage [54, 74]. By using metagenomic analysis, we provide the first evidence of specific enzymatic pathways involved in vitamin B12 metabolism whose gene abundances are potentially altered by ASP supplementation. In ASP-treated animals, we observed a significant reduction in the abundance of genes encoding vitamin B12 coenzyme biosynthesis-related enzymes and a diversification of microbial taxa contributing to these pathways. In control animals, methane- and B12-associated genes were predominantly harboured by *Prevotella* and *Methanobrevibacter*, whereas ASP-treated animals exhibited a broader taxonomic distribution of these functions, involving *Simiaoa, Starkeya, Paracoccus, Enterocloster, Dialister, Chordicoccus, Faecalibacterium*, and *Candidatus Cloacimonas*. Previous studies have shown that high ruminal concentrations of vitamin B12 are associated with increased abundance of *Prevotella* and *Methanobrevibacter*, whereas low B12 levels correlate with higher relative abundances of *Bacteroidetes, Ruminiclostridium, Butyrivibrio*, and* Succinimonas* [19, 28]. These findings support the hypothesis that ASP supplementation alters the abundance and taxonomic distribution of genes involved in vitamin B12 biosynthesis, with diversification of taxa contributing to these pathways suggesting niche displacement among microbial producers.

This observed functional redistribution may be a result of both microbial resilience and redundancy within the rumen microbiome, enabling essential processes to persist despite targeted disruption [68, 75]. Genes within the microbiome do not strictly belong to fixed taxa, instead, they can be transferred between organisms, shifting their functional contributions in response to selective forces [51]. Thus, microbiome resilience must be considered not only at the level of genomes or taxa, but also at the level of individual genes. Metagenomic assembly tools, such as the one used in this study (metaSPAdes), use k-mer abundance and read coverage in an attempt to ensure that each contig is derived from a single ‘genome’ or ‘species’ [50]. However, this is not always accurate, and some contigs can be formed from a collection of similar species and even genera in rare cases [50]. Therefore, it is plausible that certain genes that are more present in a sample than their neighbours on the same contig will have more reads mapped to them and thus provide important information on the abundance of individual genes [50]. Such functional plasticity underscores the adaptive capacity of the microbiome and may explain why efforts to reduce methane emissions through microbiome-targeted interventions can underperform, and often lose their effectiveness over time.

The potential mode of action mirrors that of known halogenated CH_4_ analogues, such as bromoform, a major active component of ASP. These compounds are known to irreversibly react with reduced vitamin B12, effectively inhibiting cobamide-dependent methyltransferases that are central to methanogenesis [12, 13, 79]. Such inhibition would disrupt the final methyl-transfer reactions in methanogenic archaea [26, 31]. Moreover, bromoform and related compounds may also impair the remethylation of homocysteine to methionine, a process likewise dependent on cobamide cofactors [6, 78]. However, while inhibition of B12-dependent methyltransferases can reduce methanogenesis, vitamin B12 is also essential for propionate formation, gluconeogenesis, methionine metabolism, and contributes to the nutritive value of ruminant-derived foods [20].

The observed decrease in glycine dehydrogenase (EC 1.4.4.2) encoding genes in ASP-treated animals may further support a disruption in C1 metabolism [29, 66]. In control animals, elevated levels/presence of glycine cleavage genes likely contributed to increased CO₂ availability, feeding hydrogenotrophic methanogenesis [81]. Changes in the abundance of genes in this pathway in treated animals may help explain the observed reduction in CH₄ production, potentially by limiting substrate availability. This is consistent with broader metabolic shifts observed in low methane-emitting ruminants. For example, proteomic analysis using SWATH-mass spectrometry in sheep with divergent CH_4_ emission phenotypes revealed differential abundance of enzymes involved in glucose and lactate metabolism, including components of the methylglyoxal pathway (a glycolytic bypass that results in D-lactate production) [5].

Similarly, genes encoding formate dehydrogenase (EC 1.17.1.9), another CO₂-generating enzyme supporting methanogenesis [30], were reduced in abundance under ASP treatment, indicating a broader suppression of formate- and glycine-derived C1 mtabolism. This finding aligns with previous work by Kelly et al. [33], demonstrating that hydrogen (H₂) and formate are critical intermediates in anaerobic ecosystems, including the rumen, where they function as electron sinks for primary fermenters when external electron acceptors are unavailable. The accumulation or depletion of these intermediates can profoundly affect fermentative balance and microbial growth. In particular, methanogens play a key role in consuming both H₂ and formate to maintain the redox equilibrium necessary for efficient fermentation [69, 71]. Experimental evidence from environments such as sewage digesters, biogas reactors, and acidic peatlands suggests that hydrogen and formate pools tend to be rapidly equilibrated and energetically interconvertible, reflecting a high degree of microbial regulation [24, 62]. In ruminants, methanogens have been shown to utilise formate alongside hydrogen and other substrates, although in situ metatranscriptomic data suggest that formate-dependent H₂ production is not a major pathway under normal conditions [21]. Nevertheless, co-culture studies demonstrate that formate can serve as a supplementary electron donor, especially when transferred from fermentative bacteria such as *Ruminococcus albus* [21]. Furthermore, methanogens such as *Methanococcus maripaludis* contain multiple formate dehydrogenase genes that redundantly support growth on formate, underscoring its central role in methanogenic metabolism [77]. Here, the observed reduction of genes encoding formate dehydrogenase under ASP treatment and therefore may represent a disruption of interspecies electron transfer pathways, particularly those feeding into methanogenesis, and could contribute to the broader inhibition of ruminal hydrogen and formate fluxes.

Additionally, our data suggest potential metabolic rerouting in ASP-treated animals. A number of genes encoding enzymes involved in acetate conversion to pyruvate, including acetate kinase (EC 2.7.2.1), were more abundant, potentially reflecting a shift in carbon flux away from acetate accumulation and CH₄ generation toward alternative energy-yielding pathways. Given that pyruvate is a key precursor for propionate synthesis [61], this shift may help explain the increased propionate (204 mmol/mol CNT vs 259 mmol/mol ASP), reduced acetate (629 mmol/mol CNT vs 552 mmol/mol ASP) and reduced CH₄ concentrations observed in the companion ASP feeding study by Krizsan et al. [35]. This is supported by previous findings from low methane-emitting ruminants, where microbial communities show lower abundances of acetate-forming enzymes, such as acetate kinase and phosphotransacetylase, and altered pyruvate metabolism routes that reduce acetyl-CoA and acetate production in favour of alternative pathways [73]. Methanogenesis inhibition is also known to increase propionate formation via hydrogen-consuming pathways, thereby mitigating hydrogen accumulation, which can otherwise impair rumen fermentation by disrupting NADH/NAD⁺ recycling and cellular redox state [7, 57]. These regulatory shifts in carbon and hydrogen flux may reflect a broader microbial adaptation to maintain redox balance and fermentation efficiency under ASP-induced suppression of methanogenesis. It is important to consider that vitamin B12 serves as an essential cofactor for propionate production via the succinate and propanediol pathways [14], whereas alternative pyruvate-derived routes operate independently of vitamin B12 [55]. Consequently, the increased propionate concentrations observed under ASP supplementation may arise from metabolic rerouting toward B12-independent pathways, together with enhanced hydrogen utilisation following suppression of methanogenesis, consistent with the observed shifts in genes involved in pyruvate metabolism.

This study employs a Latin square design, enabling robust repeated measurement across time points and providing a strong methodological foundation for detecting treatment effects within the constraints of a controlled rumen study. This study employed three animals per treatment, which, while limited in sample size, is consistent with controlled rumen studies of this nature and sufficient to generate meaningful mechanistic hypotheses. As with all metagenomic approaches, gene abundance patterns reflect functional potential rather than confirmed metabolic activity, a recognised feature of the methodology that future integration of metatranscriptomic or metaproteomic data could further resolve. The rumen microbiome is well-documented for its resilience, encompassing both resistance to and recovery from dietary perturbations [75]. A modest carryover effect was detected for enzyme commission profiles (*P* = 0.034), absent in taxonomic composition and accounting for only ~ 4% of variance, suggesting that functional recovery following exposure to bioactive compounds such as ASP may extend beyond the timeframe of taxonomic normalisation. Accordingly, future crossover designs may benefit from extended washout periods and adaptation periods to ensure full functional baseline recovery. Together, the mechanistic insights and hypotheses generated here provide a strong basis for such future work.

## Conclusions

These findings provide novel mechanistic insight into how *Asparagopsis taxiformis* supplementation modulates the rumen microbiome, highlighting effects beyond methanogen inhibition. For the first time in a metagenomic study of ASP supplementation, we identify specific enzymes involved in vitamin B12 coenzyme biosynthesis and C1 metabolism that show reduced gene abundance under ASP treatment. Alongside a shift in fermentation gene abundances towards pyruvate and propionate metabolism relative to acetate and methane production. Furthermore, through metagenomic assembly we provide taxonomic resolution of the microbial contributors to these functional shifts, computationally linking these gene abundance patterns to specific microbial taxa, a level of insight that complements culture-based approaches. Together, these findings support the potential of ASP as a strategy to mitigate ruminant methane emissions while maintaining microbial ecosystem function, and contribute to a more mechanistic understanding of how red seaweed supplementation may influence microbial ecology and metabolic potential in the rumen, with important implications for sustainable ruminant production.

## Supplementary Information

Supplementary Material 1: Taxonomy of control samples.

Supplementary Material 2: PERMANOVA carryover effects. PERMANOVA results testing the effect of previous treatment (PrevTrt) on taxonomic and functional profiles using Aitchison distances.

Supplementary Material 3: Raw reads–full lineage taxonomy, domain, KEGG pathway, EC, CAZy.

Supplementary Material 4: Alpha diversity. Mean Chao1, Shannon, and inverse Simpson indices by treatment and period, mixed-effects model results, and post-hoc contrasts for taxonomic and functional profiles. Boxplots show alpha diversity distributions across treatments and periods.

Supplementary Material 5: LEfSe results–bacteria, archaea, KEGG pathway, EC, CAZy.

Supplementary Material 6: Functional analysis. Principal coordinates analysis (PCoA) of Aitchison distance matrices based on (A) CAZy annotations, (B) EC numbers, and (C) KEGG pathways.

Supplementary Material 7: Taxonomic data assigned by Kraken2 of the contigs annotated with genes encoding enzymes of interest, data was mined using MetaPont.

## Data Availability

The datasets generated and analysed during the current study are available in the European Nucleotide Archive repository, deposited under 'PRJEB70657 ERP155573 Assessing the influence of Asparagopsis taxiformis supplementation on the rumen microbiome of dairy cows'. All code used for statistical analysis and figure creation is publicly available at github.com/lawkj/Asparagopsis_Metagenomic_analysis. The complete metagenomic workflow is publicly available at github.com/TheHuwsLab/Metagenomic_Workflow.
