# Supplementary figures and images for: Dietary inclusion of Asparagopsis taxiformis significantly reduces methane emissions in dairy cows by mechanistically altering vitamin B12-dependent and other methanogenesis precursor pathways
Source: Microbiome. 2026 Jun 12;14:171. doi: 10.1186/s40168-026-02447-0 (PMC13295230; doi:10.1186/s40168-026-02447-0)

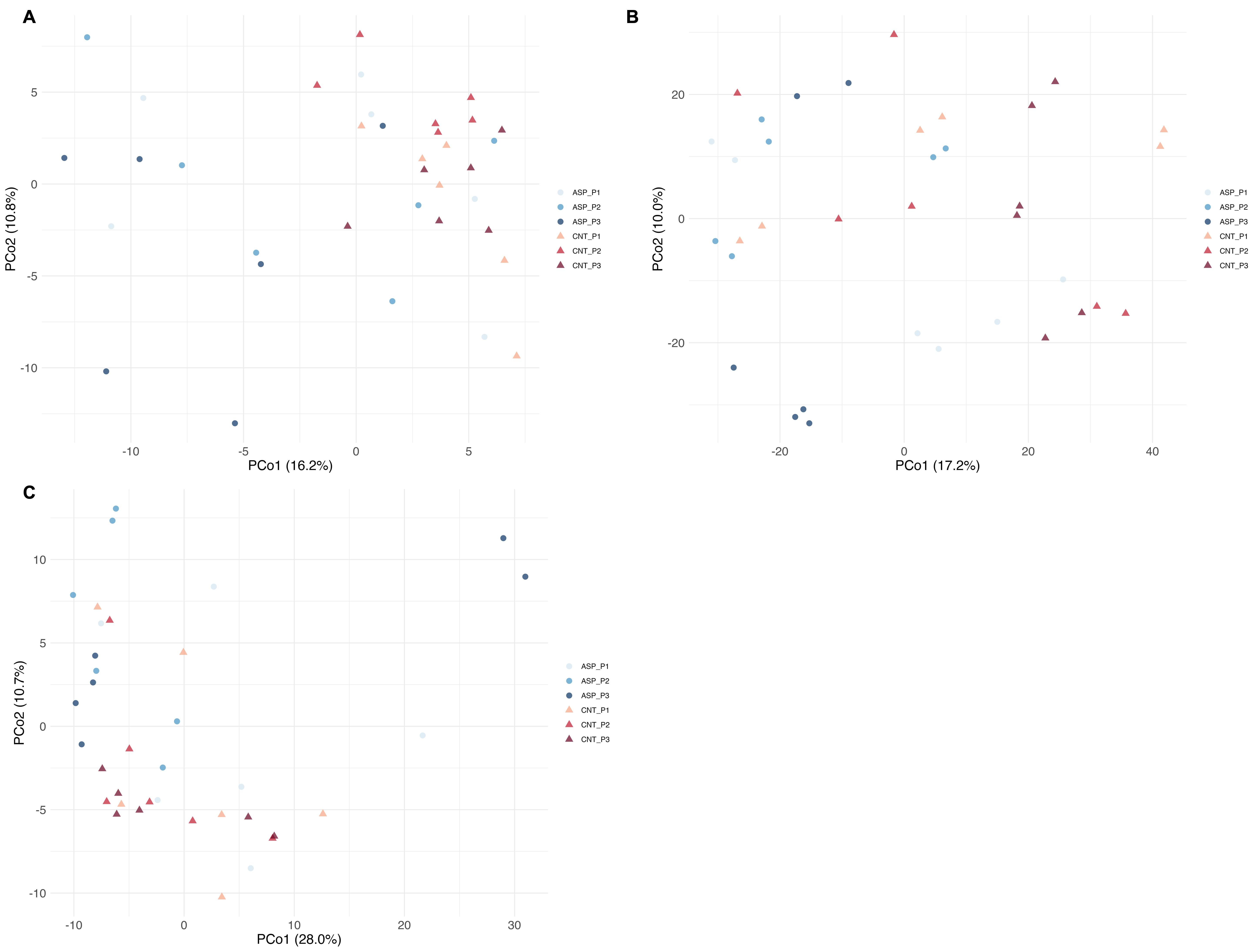

Supplement: Supplementary file 7 — Supplementary Material 6: Functional analysis. Principal coordinates analysis (PCoA) of Aitchison distance matrices based on (A) CAZy annotations, (B) EC numbers, and (C) KEGG pathways. [file 40168_2026_2447_MOESM6_ESM.png]
